# The Prevalence of Mental Problems for Chinese Children and Adolescents During COVID-19 in China: A Systematic Review and Meta-Analysis

**DOI:** 10.3389/fped.2021.661796

**Published:** 2021-10-06

**Authors:** Jiabao Chai, Huili Xu, Ning An, Pan Zhang, Fuquan Liu, Sushuang He, Na Hu, Xue Xiao, Yonghua Cui, Ying Li

**Affiliations:** ^1^Beijing Huilongguan Hospital, Peking University Huilongguan Clinical Medical School, Beijing, China; ^2^Beijing Chaoyang Center for Mental Disorder Control and Prevention, Beijing, China; ^3^Department of Psychiatry and Neuropsychology, School for Mental Health and Neuroscience, Faculty of Health, Medicine and Life Sciences, Maastricht University, Maastricht, Netherlands; ^4^Department of Psychology, Hebei Normal University, Shijiazhuang, China; ^5^Beijing Changping District Hospital of Integrated Traditional Chinese and Western Medicine, Beijing, China; ^6^Department of Psychiatry, Beijing First Hospital of Integrated Chinese and Western Medicine, Beijing, China; ^7^Department of Psychiatry, Beijing Children's Hospital, Capital Medical University, National Center for Children Healthy, Beijing, China

**Keywords:** children and adolescents, China, COVID-19, meta-analysis, depression, anxiety

## Abstract

The outbreak of coronavirus disease 2019 (COVID-19) has caused mental problems among the public and medical staff in China, especially for children and adolescents, a vulnerable group that might present with more mental problems. It seems that there is a rapid growth in the mental problems (such as depression or anxiety) of Chinese children and adolescents during the outbreak of COVID-19. Although several studies reported the prevalence of depression or anxiety problems for children and adolescents, the results are different across different age groups and sex groups. Moreover, the sample size of these studies was small. In the present study, we aim to perform a meta-analysis to identify the confirmed prevalence of depression and anxiety problems for Chinese children and adolescents during home confinement. Five databases were searched including PubMed, Web of Science, PsycINFO, Google Scholar, and the China National Knowledge Infrastructure (CNKI), and both inclusion and exclusion criteria were developed. Finally, a total of 12 studies were included in this meta-analysis. The protocol of this systematic review was registered with INPLASY (protocol ID: INPLASY202150032). It found that the pooled prevalence of mental problems was 28% (95% confidence interval, CI: 0.22–0.34), and the depression and anxiety problem for children and adolescents in China was 22% (95% CI: 0.16–0.30) and 25% (95% CI: 0.20–0.32) based on a random effect model, separately. Subgroup analysis was used to identify that there are no differences between different age groups (primary and middle school vs. high school) (*p* = 0.26). Meta-regression analysis was performed and the results showed that the moderator of boy percentage was a significant factor (*p* = 0.04). It indicated that there was an increasing number of children and adolescents with mental problems during the home confinement. It suggested that we should pay more attention to this vulnerable population during a public health crisis in the future, especially for the girls groups, and more detailed implements for mental health management were needed and should be prepared.

**Systematic Review:** The protocol of this systematic review was registered with INPLASY. The protocol ID was INPLASY202150032

## Background

In December 2019, coronavirus disease 2019 (COVID-19) broke out, thus representing another national public health emergency (1). The World Health Organization (WHO) declared that the COVID-19 outbreak was a global public health emergency on January 30, 2020 (2). The outbreak of COVID-19 has caused mental problems among the public and medical staff in China (3–5), especially for children and adolescents, a vulnerable group that might present with more mental problems (6).

For the mental problems for children and adolescents in China, there are three issues that need to be addressed. First, in our previous national survey, we have found that the prevalence of mental problems for children and adolescents was about 17% (7). But during the COVID-19 home confinement, it reported that there were nearly 30–45% of children and adolescents who might show anxiety and depression problems (8, 9). It seems that there is rapid growth for the mental problems of children and adolescents but there is a lack of more confirmed evidence. Second, due to the different stage of COVID-19, in different groups, it might show different results (6, 10). For example, a cross-sectional study among Chinese students aged 12–18 years during the COVID-19, which included 8,079 participants, reported that the prevalence of depression problem was 43.7% (8). Another survey, performed in Wuhan for the students who were restricted to home from January 23, 2020, reported the prevalence of depression problem was only 22.6% (9). It indicated that we might need more detailed reports in different dimensions of mental problems in different groups. Third, to the best of our knowledge, gender and age are potential factors affecting the mental problems of children and adolescents. But whether these factors are still the associated factors during the outbreak of COVID-19 might need further exploration.

In addition, for the reasons that account for different results of mental problems in children and adolescents, the tools used for screening might be an important associated factor (11). For example, there are at least 5 tools that were used in these related studies to assess depression in children and adolescents, which included the Chinese version of Depression, Anxiety, and Stress Scale (DASS-21) (12), the Children's Depression Inventory (CDI) (13), the Depression Self-Rating Scale for Children (DSRSC), 9 items version of Patient Health Questionnaire (PHQ-9) (14), and the Mental Health Inventory of Middle School Students (MMHI-60) which included the dimension of depression (11). But which one is more suitable for the screening for children and adolescents during the COVID-19 pandemic in China? The comparisons among these tools needed to be performed.

Therefore, in this present study, a meta-analysis will be performed to identify the pooled prevalence of mental problems including depression and anxiety problems for children and adolescents in China during the COVID-19 outbreak. The influence of age and gender on the mental problems of children and adolescents will also be explored. Furthermore, the review of screening tools for the mental health of children also will be performed which can help the future survey for researchers. In addition, we used the term “mental problem” to describe mental health related problems including depression, anxiety, stress, or other associated problems in this present study.

## Methods and Materials

### Searching of Relevant Studies

Five databases were searched including PubMed, Web of Science, PsycINFO, Google Scholar, and the China National Knowledge Infrastructure (CNKI). We only considered studies published from November 1, 2019, to March 1, 2021. The search terms were as follows: “children” or “adolescents” or “child” or “young” or “students” and “mental” or “depression” or “anxiety” or “psychological health problems” or “stress” and “COVID-19” or “coronavirus pneumonia.” References of related articles were also searched for any other relevant studies (we also searched the corresponding term in Chinese in CNKI). Due to the limited number of studies focus on sleep problems or other mental problems, we only searched related terms mentioned above. For more details of the searching strategies see Supplementary Table 1. The protocol of this systematic review was registered with INPLASY (protocol ID: INPLASY202150032).

### Inclusion Criteria and Exclusion Criteria

Both the inclusion criteria and exclusion criteria were developed as follows:

Inclusion Criteria:

1. The participants were Chinese children and adolescents;
2. The survey date was during the home confine period;
3. Have data regarding the prevalence of mental problems with a validated screening tool;
4. Written in English or Chinese;
5. COVID-19 related research.

Exclusion Criteria:

1. No data on the prevalence of mental problems were reported;
2. The assessment did not include the depression, anxiety, and stress problem;
3. The age of participants was over 18 years old (such as college students).

### Data Extraction

We extracted the following information from the included studies: authors, participants, mean ages, sample sizes, the number of boys and girls, the screening tools used, survey location, and the prevalence of the mental problem. The term “mental problem” was used to describe the mental health related problems including depression, anxiety, stress, or other associated mental problems in this present study.

### Statistical Analysis

A *p*-value < 0.05 was required to be statistically significant, and all of the analyses were performed in R (version 3.5.3) using the “meta” or “metafor” packages. A random-effects model was used to examine the pooled prevalence of depression and anxiety problems for children and adolescents (15). The *I*^2^ and forest plots were used to identify the heterogeneity of the pooled prevalence of mental problems.

First, meta-analysis was used to identify the pooled prevalence of mental problems. Second, the publication bias was tested by Egger's funnel plot. Third, the subgroup analysis (such as the different school levels) and meta-regression analyses (such as the percentages of boys) were used to explore the potential heterogeneities and identify the potential influencing factors.

## Results

### Characteristics of the Included Studies

**Figure 1:**
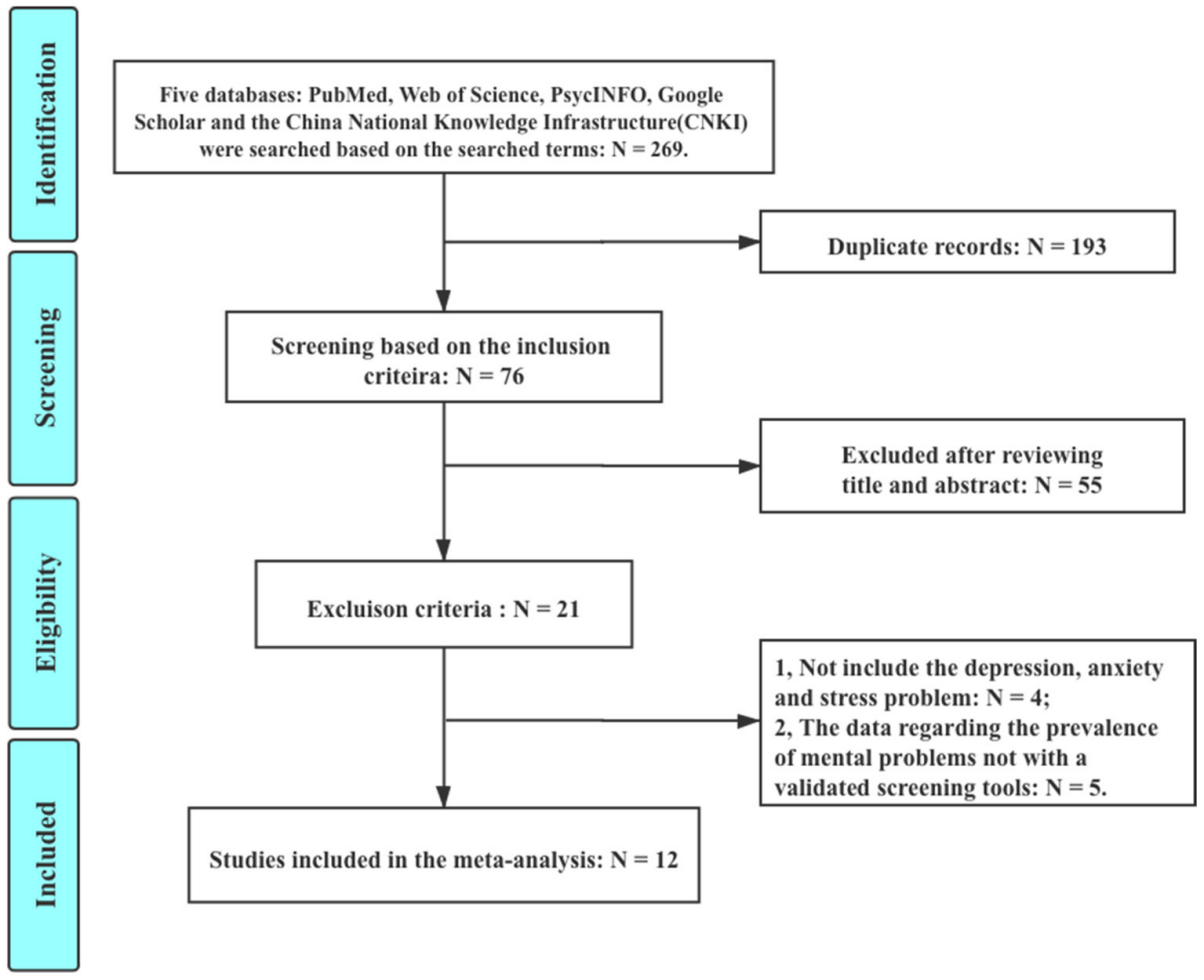
Flowchart of the identification for the included studies.

The reasons for exclusion included “duplicate records” (*N* = 193), “excluded after reviewing the title and abstract” (*N* = 55), “not include the depression, anxiety and stress problem” (*N* = 4), and “data regarding the prevalence of mental problems with a validated screening tool” (*N* = 5). Finally, a total of 12 studies included in the systematic review involved 34,276 Chinese children or adolescents (6, 8–10, 16–22). Two studies were based on the same sample (19, 20). All of them are cross-sectional studies. All surveys were conducted online due to COVID-19. For more information about the identification of included studies see Figure 1; Table 1.

**Table 1:** The included studies.

**No**.	**Study**	**Participants**	**Age (Years)**	**Sex (Boys/Girls)**	**Location**	**Screening Tools**	**Sample Size**	**Prevalence**
1	Dai et al. (17)	High school students	16.8 ± 1.1	653/836	Chengdu	DASS-21	1,399	26%
2	Liu et al. (18)	Junior high school students	Not Clear	302/535	Guangxi	SCL-90	837	22%
3	Li et al. (19)	Children and adolescents	12.82 ± 2.61	199/197	Shanxi	SCARED	396	22%
4	Wang et al. (20)	Children and adolescents	12.82 ± 2.61	199/197	Shanxi	DSRS	396	10%
5	Tang and Ying (21)	Middle school	14.01 ± 1.56	1,724/1,788	Sichuan	MMHI-60	3,512	30%
6	Wang and Xu (22)	High school	16–18	129/281	Across the country	GAD-7/PSS-10/SCSQ	410	48%
7	Zhou et al. (8)	Middle and High school	12–18	3,753/4,326	Across the country	PHQ-9/GAD-7	8,079	44%
8	Xie et al. (9)	Primary school	N/A	1,012/772	Hubei	CDI-S	1,784	23%
9	Tang et al. (16)	Primary and Secondary school	11.86 ± 2.32	2,214/2,128	Shanghai	DASS-21	4,342	25%
10	Dong et al. (10)	Primary and Middle School	12.34 ± 4.67	1,057/993	Hunan, Shandong etc.	DASS-21	2,050	18%
11	Duan et al. (6)	Primary, Secondary and High school	7–18	1,812/1,801	Across the country	CDI	3,613	22%
12	Liu et al. (3)	college and primary school students	N/A	161/238	Sichuan	SSS	399	35%

*DASS-21, Depression Anxiety Stress Scale; SCL-90, Symptom Checklist; SCARED, Screen for Child Anxiety Related Emotional Disorders; DSRS, Depression Self-rating Scale for Children; GAD-7, Generalized Anxiety Disorder; PHQ-9, Patient Health Questionnaire; MMHI-60, Mental Health Inventory of Middle-school students; PSS-10, Perceived Stress Scale; SCSQ, Simplified Coping Style Questionnaire; CDI-S, Children's Depression Inventory–Short Form; N/A, Not Available; SSS, Somatic Self-rating Scale. Prevalence, the prevalence of mental problems of each study*.

### Quality Assessment for Included Studies

Quality assessment was conducted (*N* = 12). The quality of each included study was assessed by the Joanna Briggs Institute (JBI) Critical Appraisal Checklist for Studies Reporting Prevalence Data which included 9 items (1. Was the sample frame appropriate to address the target population? 2. Were study participants sampled in an appropriate way? 3. Was the sample size adequate? 4. Were the study subjects and the setting described in detail? 5. Was the data analysis conducted with sufficient coverage of the identified sample? 6. Were valid methods used for the identification of the condition? 7. Was the condition measured in a standard, reliable way for all participants? 8. Was there appropriate statistical analysis? 9. Was the response rate adequate, and if not, was the low response rate managed appropriately?) (23). Because all studies were quantitative with a cross-sectional design, the JBI Critical Appraisal Tool for Analytical Cross-Sectional Studies was used. Studies were appraised as having low, moderate, or high methodological quality. There are two authors (JC and HX) who independently assessed each included study and reached a consensus on any differences. For more details see Table 2. The percentage of Yes items was calculated. If this percentage of Yes item is more than 75%, the study was included (23, 24).

**Table 2:** The JBI Critical Appraisal Checklist for Studies Reporting Prevalence Data.

**Studies**	**1. Was the sample frame appropriate to address the target population?**	**2. Were study participants sampled in an appropriate way?**	**3. Was the sample size adequate?**	**4.Were the study subjects and the setting described in detail?**	**5. Was the data analysis conducted with sufficient coverage of the identified sample?**	**6. Were valid methods used for the identification of the condition?**	**7.Was the condition measured in a standard, reliable way for all participants?**	**8. Was there appropriate statistical analysis?**	**9. Was the response rate adequate, and if not, was the low response rate managed appropriately?**	**Y%**
Dai et al. (17)	Yes	Yes	Yes	Yes	Yes	No	Yes	Yes	Yes	88.9%
Liu et al. (18)	Yes	Yes	Yes	Yes	Yes	No	Yes	Yes	Yes	88.9%
Li et al. (19)	Yes	Yes	Not Clear	Yes	Yes	Yes	Yes	Yes	Yes	88.9%
Wang et al. (20)	Yes	Yes	Not Clear	Yes	Yes	Yes	Yes	Yes	Yes	88.9%
Tang and Ying (21)	Yes	Yes	Yes	Yes	Yes	Yes	Yes	Yes	Yes	100%
Wang and Xu (22)	Yes	Yes	Not Clear	Yes	Yes	Yes	Yes	Yes	Yes	88.9%
Zhou et al. (8)	Yes	Yes	Yes	Yes	Yes	Yes	Yes	Yes	Yes	100%
Xie et al. (9)	Yes	Yes	Not Clear	Yes	Yes	Yes	Yes	Yes	Yes	88.9%
Tang et al. (16)	Yes	Yes	Not Clear	Yes	Yes	Yes	Yes	Yes	Yes	88.9%
Dong et al. (10)	Yes	Yes	Yes	Yes	Yes	Yes	Yes	Yes	Yes	100%
Liu et al. (3)	Yes	Yes	Yes	Yes	Yes	Yes	Yes	Yes	Yes	100%

*Y%, percentage of Yes items*.

### The Pooled Prevalence of Mental Problems for Children and Adolescents

**Figure 2:**
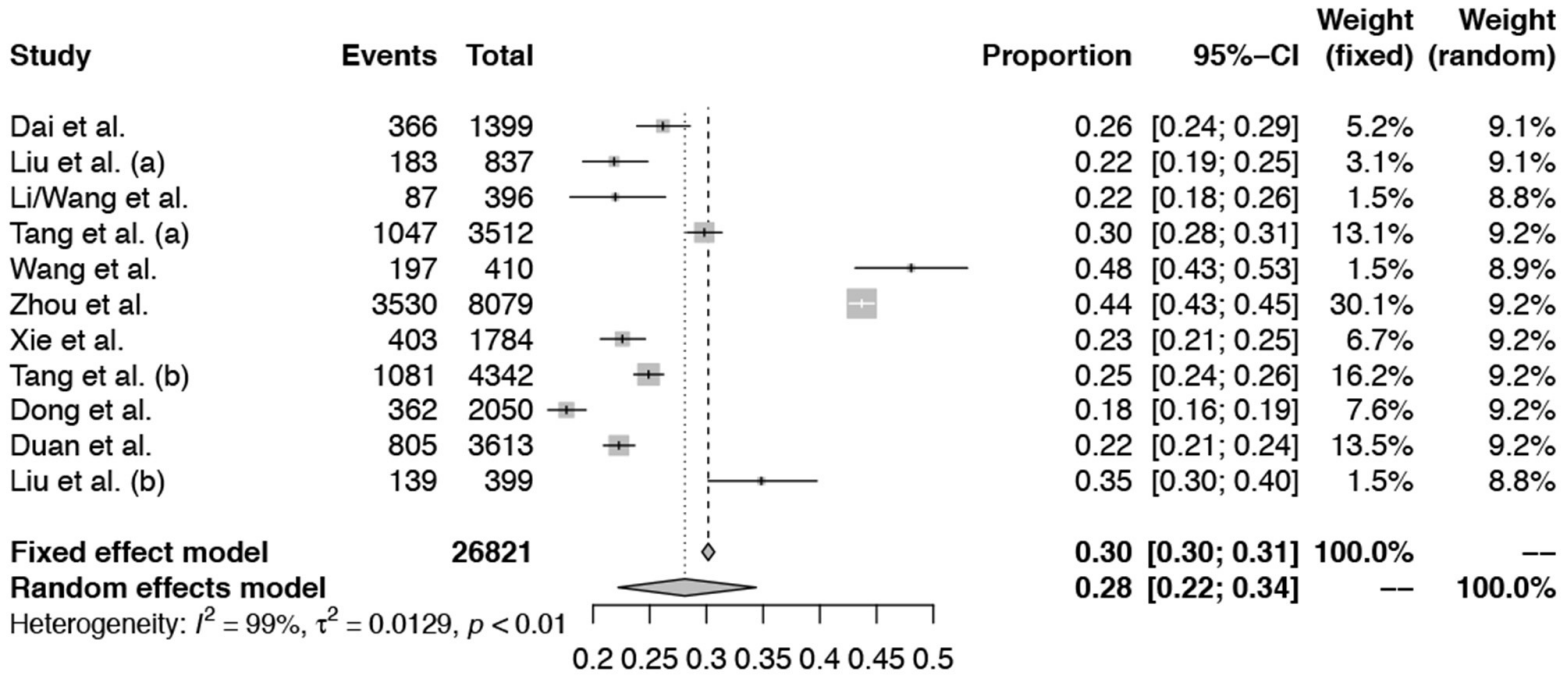
Forest plots of the meta-analysis for the pooled prevalence of the mental problem.

**Figure 3:**
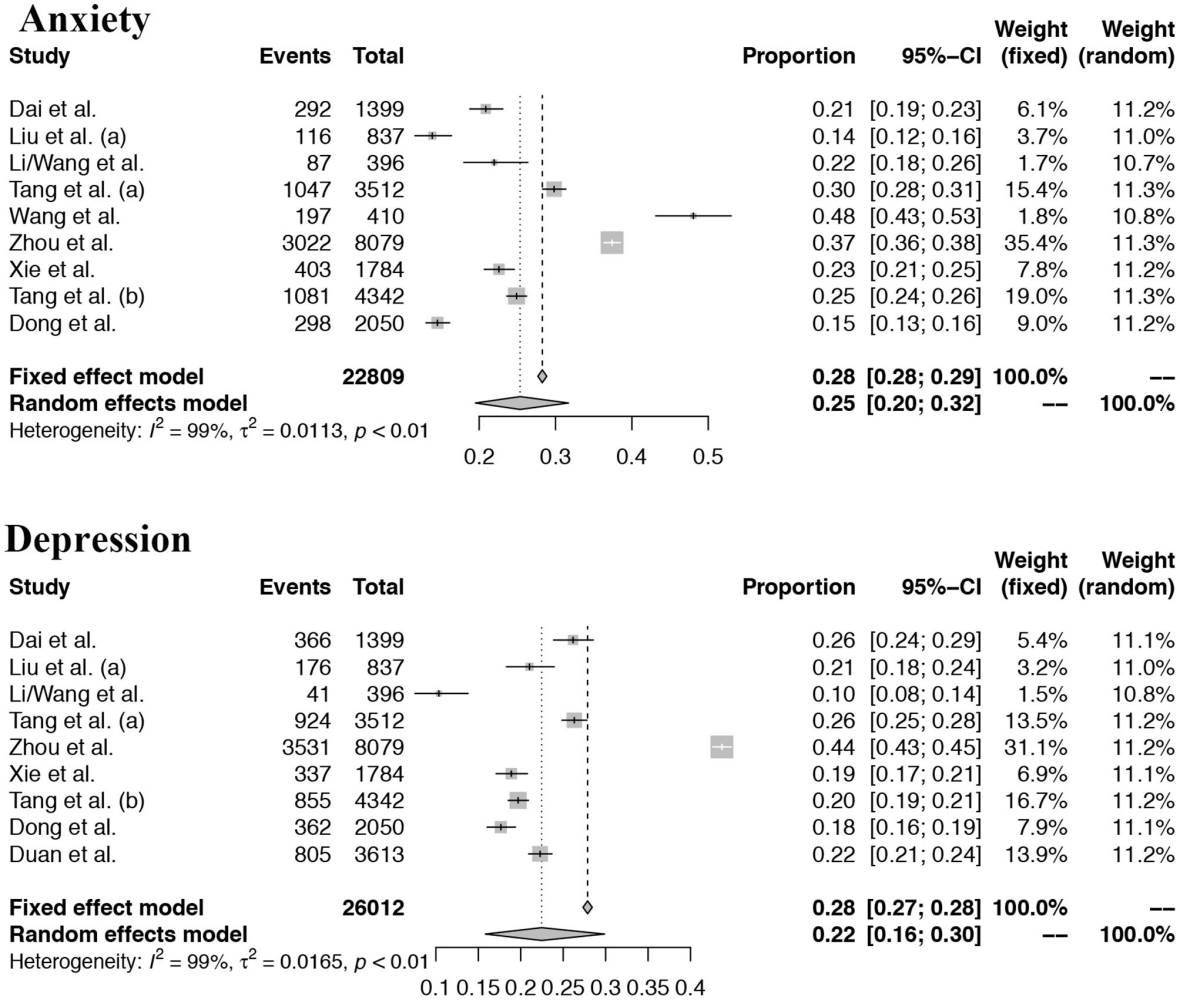
Forest plots of the depression and anxiety problems.

For the mental problems (including depression problems or the anxiety problem, or stress problems), we found that the pooled prevalence of mental problems was 28% (95% confidence interval, CI: 0.22–0.34), and the depression and anxiety problem for children and adolescents in China was 22% (95% CI: 0.16–0.30) and 25% (95% CI: 0.20–0.32) based on a random-effect model, separately. For more details see Figures 2, 3.

### Publication Bias

Egger's funnel plot was used to identify the publication bias, and we found that no publication bias was identified (*p* = 0.26). For more details see Supplementary Figure 1. Sensitivity analysis was performed to explore the heterogeneity of the pooled prevalence of mental problems. But no study showed the change of the heterogeneity more than 5%. For more details see Supplementary Figure 2.

### Subgroup Analysis and Meta-Regression Analysis

**Figure 4:**
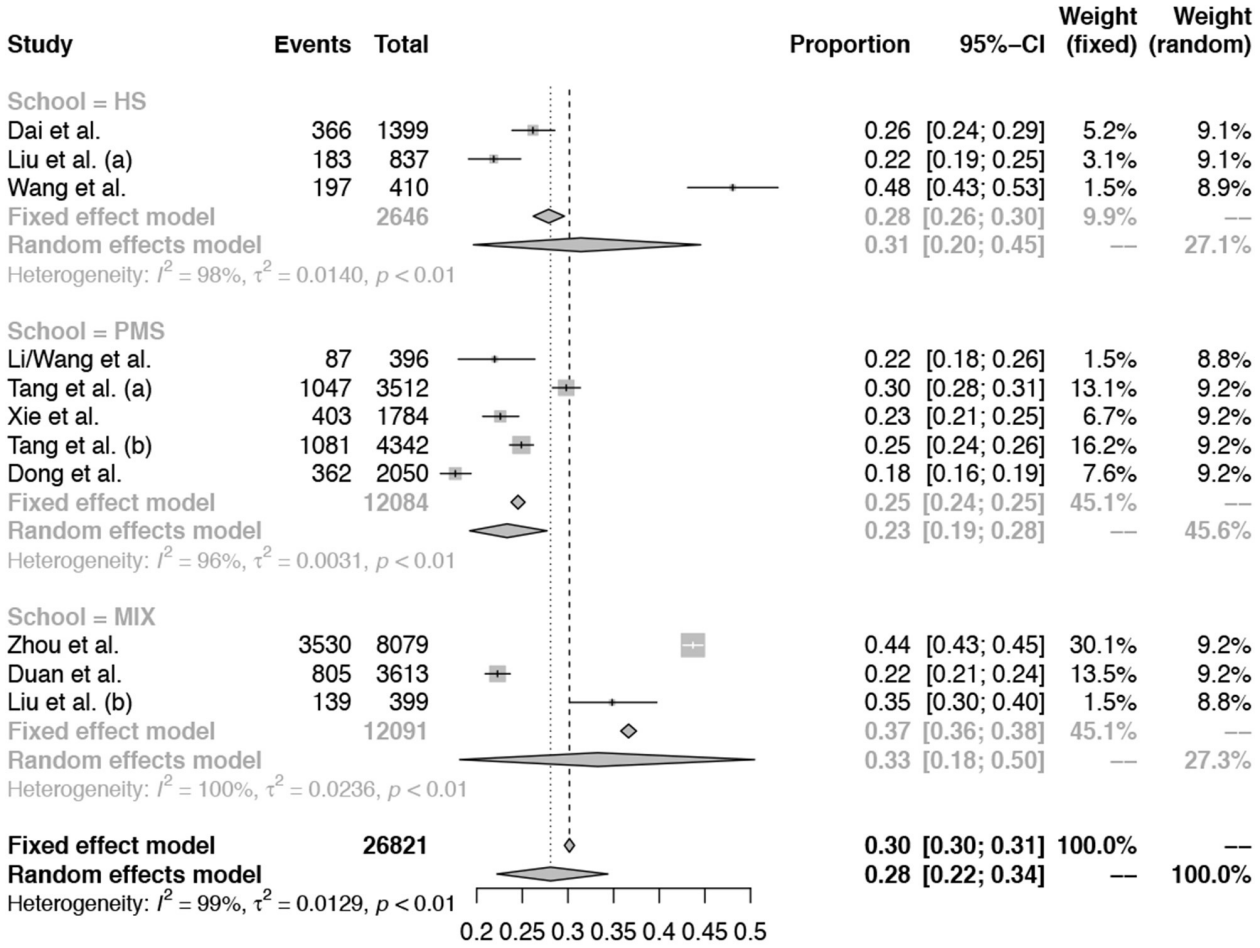
Subgroup analysis by different “age groups” (primary and middle school vs. high school).

**Figure 5:**
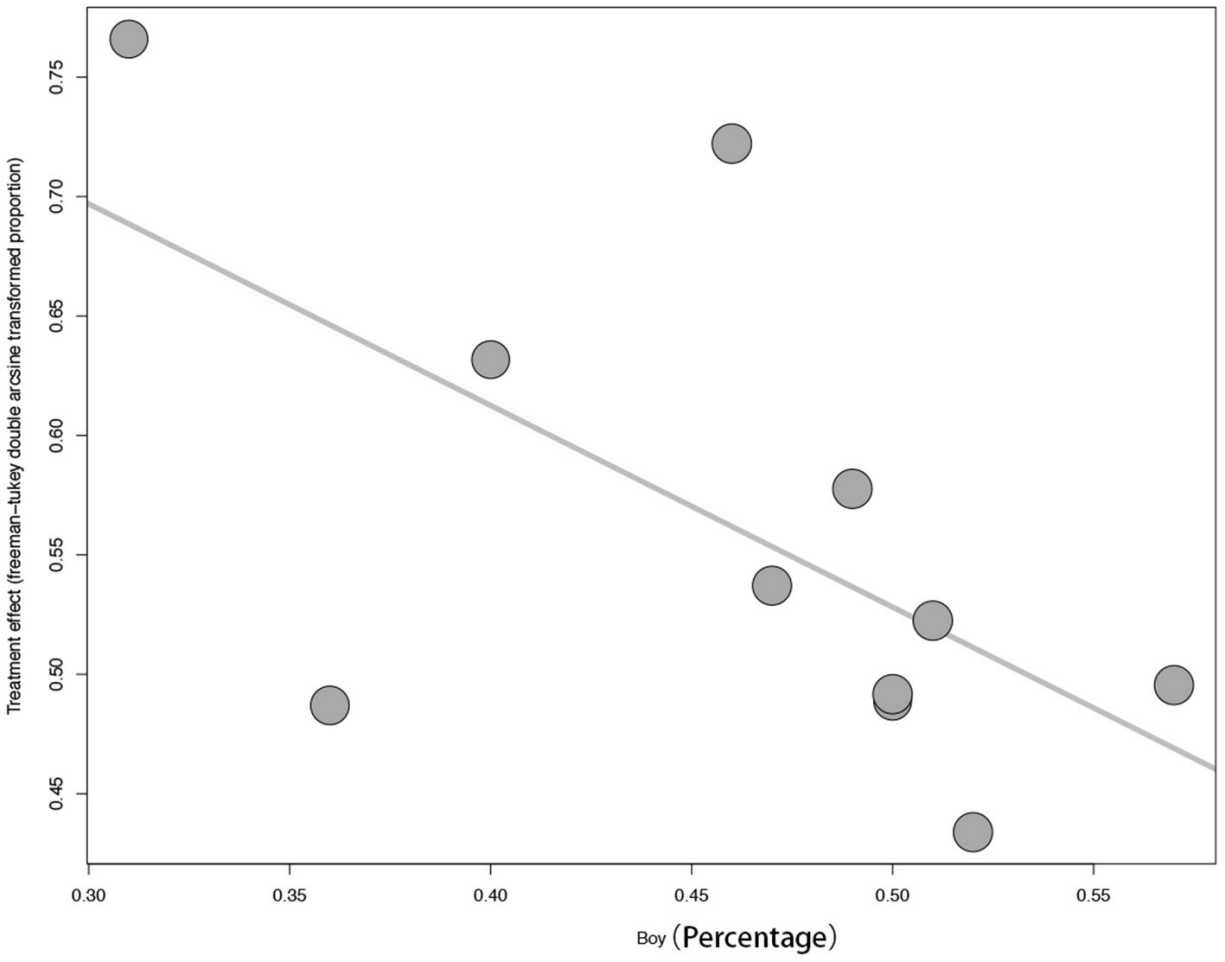
The meta-regression analysis by “sex.”

Subgroup analysis was performed by different age groups (primary and middle school vs. high school). For more details see Figure 4. But there are no differences identified between different age groups. Meta-regression analysis was performed and the results showed that the moderator of boy percentage was a significant factor (*p* = 0.04). For more details see Figure 5.

### The Summary of Screening Tools in the Included Studies

In addition, we summarize the related tools for the mental health of children and adolescents used in China: the number of items, the cutoff scores, the minimal age suitable for each tool, as well as the main strength and limitation for this tool. For more details see Table 3. A total of nine scales were used in the included studies to screen for depression or anxiety. Three items of DSSS-21 were used, two things of GAD-7 and Scared were used, and the other seven scales were used once each. It showed that the DASS-21 was used more frequently than other tools.

**Table 3:** Screening tools for anxiety and depression.

**Scale**	**Items**	**Minimum Age**	**Cutoff**	**Strength and Limitation**
DASS-21	21	11	10 for anxiety 10 for depression 15 for stress	Dimension included depression, anxiety, and stress, but not suitable for individuals aged <10 years;
SCL-90	90	8	Factor score ≥ 2	Comprehensive tool for mental health but with too much items, not suitable for screening assessment;
SCARED	41	8	14–16	Assessing different dimensions for Anxiety but with a little too much items;
DSRSC	18	7	14/15	Assessing different dimensions for Depression;
GAD-7	7	10	5–9 and above	Screening tool for Anxiety symptoms but only include the general anxiety symptoms
PHQ-9	9	11	5–9 and above	Screening tool for Depression symptoms but not suitable for children <10
MMHI-60	60	10	Factor score ≥ 2	Comprehensive tool for mental health but with too much items
CDI	27	7	Clinical: 19 Subclinical: 12–18	Assessment the core features for Depression symptoms;
SSS	20	N/A	Positive ≥40	Assess physical, anxiety, depression, and mixed anxiety and depression symptoms.

*DASS, Depression Anxiety Stress Scale; SCL-90, Symptom Check-list 90; SCARED, Screen for Child Anxiety Related Emotional Disorders; GAD-7, Generalized Anxiety Disorder 7; PHQ-9, 9 items of Patient Health Questionnaire; DSRSC, Depression Self-rating Scale for Children; MMHI-60, Mental Health Inventory of Middle School Students; CDI, Children's Depression Inventory; SSS, Somatic Self-rating Scale*.

## Discussion

In this present study, we found that the pooled prevalence of mental problem was 28%. For depression problem, it was 22% and the anxiety problem was 25%, while the mental problems in our previous national survey were only 17% (7). It indicated that the prevalence of mental problems of children and adolescents was increasing during home confinement after the outbreak of COVID-19. After the COVID-19 outbreak, nearly all schools were shut down nationally in China and nearly all students had to face home confinement (25). Although online teaching activities were provided to children and adolescents during this period (26, 27), it seemed that mental health-protecting activities were also needed. A survey conducted in the United States found that 40.1% of parents reported observing signs of distress in their children, which seemed higher than in China. Recently, a rapid systematic review of the mental problem showed that children and adolescents are more likely to suffer a high prevalence of depression and anxiety problem during a pandemic (28). The impact of the pandemic on the mental health of children and adolescents is inevitable.

In this present study, we also found that sex might be an influencing factor for the mental problem of children and adolescents. For the influencing factor of sex, it was also confirmed by a study from the United States which included 683 adolescents (29). But negative results were also reported (9). Moreover, one study found higher mental problems in older children (8), but negative results were also found in this present study. To date, there are a strikingly small number of published studies examining the prevalence of mental problems in children and adolescents during the COVID-19. Therefore, a larger number of studies were needed. In the future, we should further explore more the protective factors and risk factors that might influence the mental problems of children and adolescents.

Children facing unexpected and unknown events typically exhibit various stress reactions. Several studies have documented the damaging effects of psychological stress due to negative events in children (30, 31). For the increasing mental problems for children and adolescents during COVID-19 in China, several reasons might account for this phenomenon. Firstly, during the course of home confinement, they are often forced to stay home for long periods, which might result in limited connection with their friends and reduced outdoor activity (32, 33). Secondly, it has been found that during an epidemic outbreak, the adults might also experience negative emotional responses (34). For example, it found that COVID-19 causes moderate-to-severe mental health in about one-third of adults for Chinese people (4). When these adults with depression or anxiety symptoms faced their children at home, conflict might more easily occur (35). Thirdly, a recent report on mitigating the effects of home confinement on children stated that home confinement could offer an excellent opportunity to work on and improve interactions between parents and children (25). However, when parents have to take the roles of both caregiver and teacher during home confinement for a long time, there can be conflict. It indicated that the relationship between the parents and their children plays a critical role in children's mental health, and with the outbreak of COVID-19 in China, this relationship is facing further challenges (36). Finally, after the outbreak of COVID-19, information overload often happened for children and adolescents which might lead to more negative emotions with them (37). In addition, many Chinese parents tend to overlook their children's mental health. For example, due to the lack of knowledge about children's mental health, many parents cannot differentiate normal and abnormal behavioral and emotional problems in their children, especially for the left-behind children (parents go to the big city to work, not around the child, and the child is taken care of by grandparents or grandparents) (38, 39). Overall, the public awareness of the importance of mental health for children and adolescents during public health crises still needs to be strengthened.

How to protect children and adolescents from the negative impact on mental health during a public health crisis such as COVID-19? Several issues need to be addressed in the future. First is to strengthen the public awareness about mental health for children and adolescents. Second, regular daily activities management and a fixed date to contact their friends and teachers online might ease their stress during social distancing. Third, a “soft” parent-child relationship that communicates in a more friendly, sincere, and democratic way was needed. Finally, limited time usage of cell phones or pads is also necessary for children and adolescents to avoid information overload. As the pandemic continues, we should monitor the impact on children's and adolescents' mental problems and help them to improve their mental health outcomes.

In addition, for the tools used for screening the depression and anxiety of children and adolescents in China, the DASS-21 might be the suitable tool for this usage (including the dimension of depression, anxiety, and stress). GAD-7 and PHQ-9 can be used together for the quick screening of depression and anxiety. For more confirmed assessment of depression or anxiety and associative symptoms for children and adolescents, SCARED and DSRSC were recommended. The MMHI-60 can be used as a comprehensive tool for screening mental problems in Chinese children and adolescents.

Two limitations need to be addressed. First, the high heterogeneity of the meta-analysis results in the present study indicated that we should identify the potentially influential factors which might be associated with these mixed results in future. The most possible reason for this study is the limited number of studies, different regions, the different tools for screening, and the date of the survey. With the accumulated data in the future, we should pay more attention to these factors. It should be noted that the “regions” might be one of the most important variables which need to be explored. However, we could not classify the “region” in suitable terms. Some of the surveys were based on the national regions or two regions which lead to the difficulty to perform the subgroup analysis. Second, we only identified the pooled prevalence of depression and anxiety in children and adolescents; other mental health problems, such as posttraumatic stress disorder (PTSD), sleep problems, and appetite problems, should also be explored. In addition, there is a lack of screening tools for children younger than 8. In future studies, we should develop more validated tools for different age groups.

## Conclusions

In this present study, we found that the pooled prevalence of mental problem was 28%. For the depression problem, it was 22% and the anxiety problem was 25%. It indicated that there was an increasing number of mental problems in this special period for children and adolescents. We found that sex might be an influencing factor for the mental problems of children. It suggested that we should pay more attention to this vulnerable population during a public health crisis in the future, and more detailed implements for mental health management for this vulnerable population were needed.

## Data Availability Statement

The original contributions presented in the study are included in the article/Supplementary Material, further inquiries can be directed to the corresponding author/s.

## Author Contributions

For this manuscript, YC and YL took the initiative. JC, NH, FL, and SH finished the data extraction. PZ and NA performed the data analysis. JC, XX, and HX finished the draft. All authors contributed to the article and approved the submitted version.

## Funding

This study is supported by the National Natural Science Foundation of China, No. 82001445. YL was the founder. The funding body had no further role in the study design, the collection, analysis, and interpretation of data, the writing of the manuscript, and the decision to submit the paper for publication.

## Conflict of Interest

The authors declare that the research was conducted in the absence of any commercial or financial relationships that could be construed as a potential conflict of interest.

## Publisher's Note

All claims expressed in this article are solely those of the authors and do not necessarily represent those of their affiliated organizations, or those of the publisher, the editors and the reviewers. Any product that may be evaluated in this article, or claim that may be made by its manufacturer, is not guaranteed or endorsed by the publisher.

## Supplementary Material

Supplementary Figure 1 The funnel plot of the meta-analysis.

Supplementary Figure 2 The sensitivity analysis for the included studies.
