# Supplementary figures and images for: The Prevalence of Mental Problems for Chinese Children and Adolescents During COVID-19 in China: A Systematic Review and Meta-Analysis
Source: Front Pediatr. 2021 Oct 6;9:661796. doi: 10.3389/fped.2021.661796 (PMC8527981; doi:10.3389/fped.2021.661796)

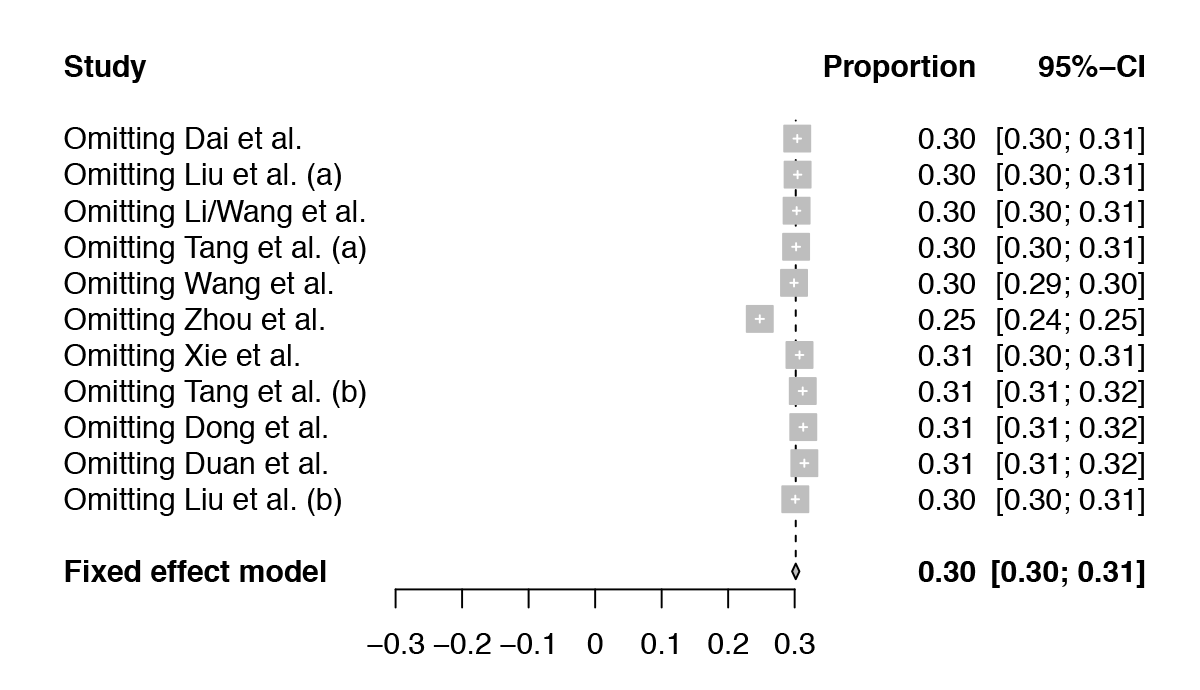

Supplement: Supplementary Figure 1 — The funnel plot of the meta-analysis. [file Image_1.TIF]

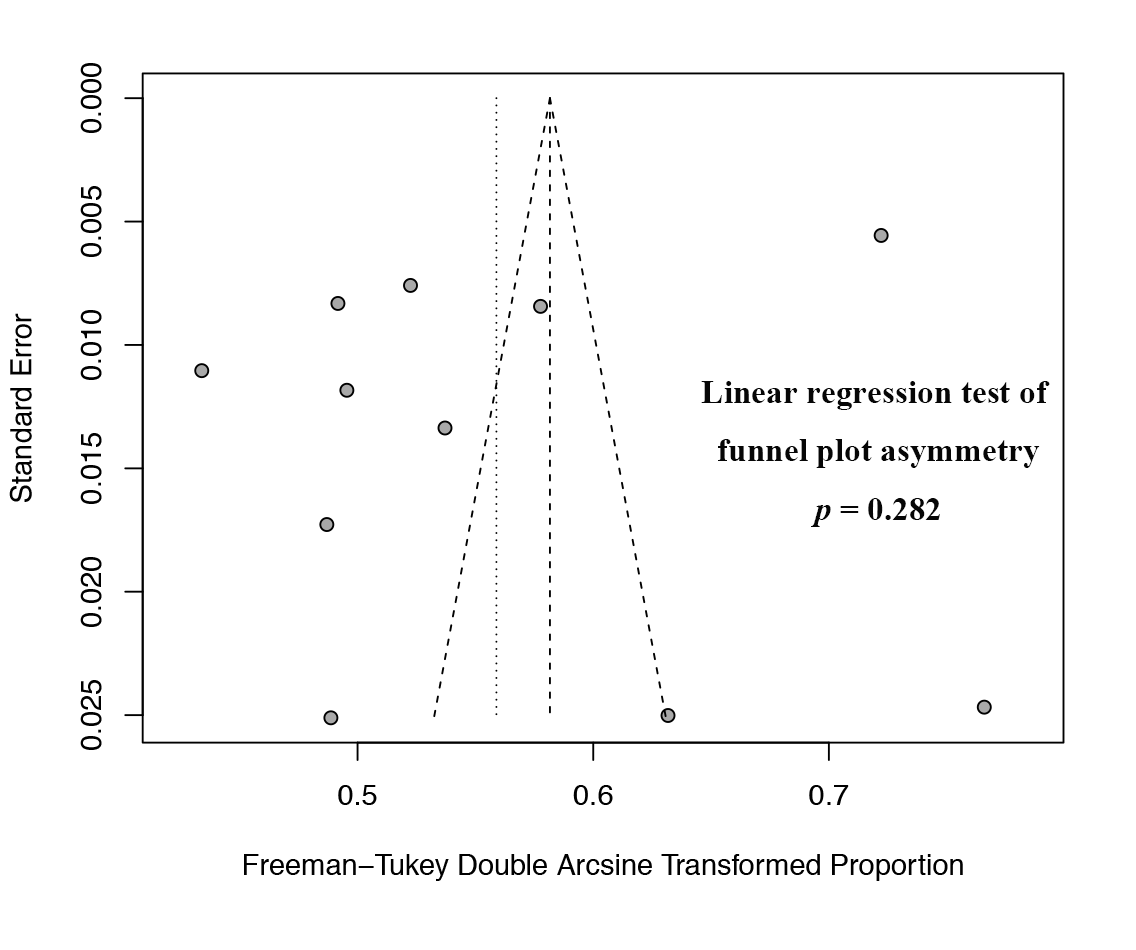

Supplement: Supplementary Figure 2 — The sensitivity analysis for the included studies. [file Image_2.TIF]
