# Supplementary material for: The Prevalence of Mental Problems for Chinese Children and Adolescents During COVID-19 in China: A Systematic Review and Meta-Analysis
Source: Front Pediatr. 2021 Oct 6;9:661796. doi: 10.3389/fped.2021.661796 (PMC8527981; doi:10.3389/fped.2021.661796)
Supplement: Supplementary file 3 [file Table_1.docx]

**Supplementary Table 1 (Searching terms and searching strategies)**

| **Searching terms**  **“Or” used between these terms** |  | **Searching terms**  **“Or” used between these terms** |  | **Searching terms**  **“Or” used between these terms** |
| --- | --- | --- | --- | --- |
| **children** | **And** | **mental** | **And** | **COVID-19** |
| **child** |  | **depression** |  | **coronavirus pneumonia** |
| **adolescents** |  | **anxiety** |  | **-** |
| **students** |  | **psychological health problems** |  | **-** |
| **young** |  | **stress** |  | **-** |
